# Awareness on psychosomatic health among adolescent girls of three schools in north Kolkata

**DOI:** 10.4103/0019-5545.74312

**Published:** 2010

**Authors:** Palas Das, Ranabir Pal, Shrayan Pal

**Affiliations:** Department of Community Medicine, Midnapore Medical College, West Bengal, India; 1 Sikkim Manipal Institute of Medical Sciences (SMIMS) and Central Referral Hospital (CRH), 5^th^ Mile, Tadong, Gangtok, Sikkim - 737 102, India

**Keywords:** Adolescent, attitude, knowledge

## Abstract

**Introduction:**

Psychosomatic health of adolescent girls at crossroads of childhood and mature adulthood, may lead to various health problems in future.

**Objective:**

To determine the improvement in the knowledge and attitude on health among adolescent girl students of Kolkata after the health education intervention.

**Materials and Methods:**

This ‘Health Education Intervention Study’ was conducted in October and November 2006, in three senior secondary schools of North Kolkata. The Simple Random Sampling Technique was applied to select three schools from the spot map of North Kolkata for this study, and 282 girl students in the adolescent age group of 13 to 19 years were selected from the completed updated list of students from the enrollment registers in these schools.

**Results:**

The mean age of the participants was 15.7 years (±1.8 years). This health education intervention showed a significant improvement in their knowledge on adolescent health, in the aspects of sex differences in pubertal spurts, probable causes of health problems during adolescence, physical changes in adolescent boys and girls, and psychological problems of adolescence. A significant improvement in positive attitude was observed, with regard to their opinion on substance abuse in the adolescent period and importance of sex education for adolescents.

**Conclusion:**

This study revealed some unknown parts of psychosomatic health among adolescent girls, in this part of India.

## INTRODUCTION

In the era of the pandemic of HIV / AIDS, much attention has been paid to migratory laborers and bridge population, because of their overt risky behavior, although little attention has been paid to other parts of the population. HIV / AIDS is increasing at a faster rate globally in the early adult population. People in developing countries like India are sitting on the fence of a conservative Indian Society and the sexually liberated west. Moreover, as they are at the crossroad of childhood and mature adulthood, many diversions come in the way of their rational thinking. They go for experimentation of different types. Any significant difference in their perception with regard to physical and mental development in their own body also needs to be explored.

The state of sex education programs in Asia is at various stages of development. Indonesia, Mongolia, and South Korea have a systematic policy framework for teaching the facts of sex within schools. Malaysia, the Philippines, and Thailand have assessed adolescent reproductive health needs with a view to developing adolescent-specific training, messages, and materials. India has programs aimed at children from the age of nine to sixteen years. In India, there is a huge debate on the curriculum of sex education and when should it be increased. Attempts by state governments to introduce sex education as a compulsory part of the curriculum have often been met with harsh criticism by political parties, who claim that sex education ‘is against Indian culture,’ and would mislead children. Bangladesh, Myanmar, Nepal, and Pakistan have no coordinated sex education programs. The International Planned Parenthood Federation and the BBC World Service ran a 12-part series known as Sexwise, which discussed sex education, family life education, contraception, and parenting. It was first launched in South Asia and then extended worldwide.[1] This study intended to verify the desired modification level, after health education intervention, in female students in the adolescent age groups in Kolkata.

## MATERIALS AND METHODS

### Study design

Health Education Intervention Study.

### Study period

Two months (October and November 2006).

### Study area

Three senior secondary Schools of North Kolkata.

### Sampling technique

The Simple Random Sampling Technique was applied to select three schools from the spot map of North Kolkata for this study.

### Study population

Two hundred and eighty-two female students in the adolescent age group of 13 – 19 years, who were studying in these schools, participated in this study.

### Selection criteria

All the adolescent girl students belonging to the age group of 13 years and 19 years were selected for this study.

### Inclusion criteria

All adolescent girls studying in Classes X, XI, and XII, and belonged to the age group of 13 – 19 years, and who gave informed verbal consent to participate in the study and were present on the day of the pre-test, were included in this study.

### Exclusion criteria

All adolescent girl students, who were absent during the pre-test session and also the participants who attended the pre-test session, but failed to appear for subsequent post-test sessions were excluded from this study.

Content Validity and Reliability of Study Instruments: The Health Education module on Adolescent Health was related with the sociodemographic situation prevailing in India. It was developed on the information provided in the Reproductive and Child Health (RCH) Module for Medical Officers, published by the Ministry of Health and Family Welfare. The experts of Community Medicine and Obstetrics and Gynecology (OBG) assessed the content validity of the Health Education Module by pre-testing on a randomly selected sample of 20 adolescent female students of a different senior secondary school (not included in this study), following which some of the questions from the interview schedule were modified prior to the study, for ensuring feasibility, acceptability, time management, and reliability. By initial translation, back-translation, and re-translation, followed by a pilot study, the questionnaire was custom-made for the study. Pre- and Post-test questionnaires on the knowledge and attitudes with regard to adolescent health were used to collect the data. Audio-Visual teaching materials and handouts were used during the health education intervention.

### Data collection procedure

All the participants were explained about the purpose of the study and were ensured strict confidentiality, following which verbal informed consent was taken from each of them before the interview. The participants were given the option of not participating in the study if they did not want to. Then using the interview technique the data were collected. Initially, all the participants were evaluated by the pre-test questionnaire. Then health education intervention was organized in multiple sessions, each lasting for two hours, spread over a period of two weeks, for batches of 20 students each, by specialists in Community Medicine. After the end of each of these sessions, the first post-test questionnaire was administered. A second post-test was applied after four weeks and the difference in knowledge and change in attitude was noted. To increase the participatory response, this health education intervention program was followed by a clinical examination by Community Medicine and OBG specialists. Referral services and medication were provided free of cost to the needy participants. A pre-tested closed ended questionnaire was used to assess the impact of health education intervention, where a score of ‘1’ was given to incorrect knowledge and negative attitude, while a score of ‘2’ was given to correct knowledge and positive attitude.

### Data analysis

The data collected were thoroughly cleaned and entered into Excel spread sheets, and analysis was carried out using the statistical package SPSS (Statistical Package for Social Sciences) version 10.0 for Windows. The procedures involved were transcription, preliminary data inspection, content analysis, and interpretation. The results were calculated in terms of proportions. Proportions were used to derive information on the baseline characteristics of the participants. The paired t-test was applied to the pre-test and the second post-test results of Knowledge and Attitude on Adolescent Health, to assess the impact of health education intervention. *P* value<0.05 was considered as statistically significant.

## RESULTS

The mean age of the participants was 15.7 years (±1.8 years). The majority of respondents belonged to the Hindu community (76.7%), from the Middle / High Socioeconomic Status (78.9%). A majority of the participants had poor knowledge and negative attitude regarding Adolescent Health, before the health education intervention.

Significant improvement in Knowledge and Positive Attitude between pre- and post-test results was observed regarding the following aspects:

- Significant Improvement in the Knowledge on Adolescent Health was observed with respect to the following aspects: (a) sex differences in pubertal spurts in adolescents, (b) probable causes of health problems in the adolescent period, (c) physical changes in adolescent boys, (d) physical changes in adolescent girls, and (e) psychological problems in the adolescent period.
- Significant Improvement in Positive Attitude on Adolescent Health was observed with respect to the following aspects: (a) opinion on substance abuse in the adolescent period, (b) importance of sex education for adolescents [Table 1].

**Table 1:** Knowledge and attitude on psychosomatic health among adolescent girls of Kolkata

Questions (*n* = 282)	Pre-test (%)	Post-test (%)		
Knowledge 1. Sex differences in pubertal spurts of adolescence	71 (25.18)	241 (85.46)
Knowledge 2. Adolescent period starts at age	221 (78.37)	242 (85.82)
Knowledge 3. Probable causes of health problems in the adolescent period	91 (32.27)	245 (86.88)
Knowledge 4. Physical changes in adolescent boys	134 (47.52)	281 (99.64)
Knowledge 5. Physical changes in adolescent girls	148 (52.48)	259 (91.84)
Knowledge 6. Frequently, during the adolescent period, deficiency of vitamins occurs	199 (70.57)	233 (82.62)
Knowledge 7. Psychological problems of the adolescent period	160 (56.74)	259 (91.84)
Attitude 1. Why adolescents are addicted to tobacco, drugs, alcohol, and so on	35 (12.41)	199 (70.57)
Attitude 2. Importance of education on sexual medicine and sexuality	61 (21.63)	195 (69.15)

The health education module on Adolescent Health was well accepted by the adolescent female student community. The participatory response and acceptance of this program was more spontaneous due to the fact that this health education intervention program was followed by clinical examination by the Community Medicine and OBG specialists, with provision of free referral services and medication to the needy participants.

## DISCUSSIONS

Our study revealed a positive impact on the ‘Knowledge’ and ‘Positive Attitude’ between pre-test and post-test results. A great majority of the participants had poor knowledge and a negative attitude regarding adolescent psychosomatic health before the health education intervention program. Although some sort of sex education is part of many schools’ curriculum, it remains a controversial topic in several countries. How much and at what age should schoolchildren be taught about contraception or safer sex and masturbation? Moreover, whether moral education should be included or excluded is debatable. Incidentally we are yet to find any similar published study from India to compare with our findings. During 1993 – 1994, five sex information programs were conducted in various secondary schools in Rome, involving a total of 292 students, whose ages ranged from 14 to 21 years. The courses were organized in five sessions, lasting two hours each, and were held by a specialist in obstetrics and gynecology. Visual teaching materials were presented, followed by discussions. The initiative was evaluated by means of three questionnaires: the pre-test questionnaire on attitudes and knowledge about reproductive health; the second was administered at the end of the course, about recommendations; and the third one verified the modification of the knowledge level four months later. Twenty to fifty percent of the students answered all the questions about reproductive health correctly before the course, and 70 – 100% of them at the end of four months. Ninety-five percent of the sample thought that the school should provide information about sexuality and 74% of the students suggested that it should be introduced in the lower grades of secondary schools. Eighty-seven percent of the subjects knew that the condom also offered protection against sexually transmitted diseases. Family counseling should focus on prevention activities for the school-age population.[2]

In a study to investigate the knowledge of sexually transmitted infections (STI) among women, of age between 15 and 49 years, in a rural district of North Vietnam, and to evaluate the possible association between socioeconomic factors and STI knowledge, the researchers concluded that the low levels of STI knowledge among the study respondents reflected the potential importance of health education interventions, to improve STI knowledge in the general population. Young and unmarried women, the most vulnerable population, must be specifically targeted. Intervention programs should be diversified and tailor-made for each group.[3]

Little emphasis on educational and other efforts to prevent infection occurring in the first place is one of common reasons why STI control programs often failed in the low-income countries.[4]

Researchers on HIV in India also concluded that education is essential to assist young people to make informed decisions about their sexual health.[5] Some areas still needed special attention. In a study conducted among school children in the state of Haryana, India, 57% believed that persons with HIV / AIDS could be detected by their physical appearance.[6]

Although contemporary literature reveals that oral contraceptives are safe for adolescents, in India, less than 10% of the adolescents use any form of contraceptive.[7]

In order to intensify the focus on STI prevention among young people, in June 2005, the Government of India announced the National Adolescent Education Program (AEP). While the main focus of the program was on HIV / AIDS prevention, it also covered sexual reproductive health issues, gender, and life skills.[8]

The review on HIV in India concluded that in a conservative society where sex-related issues constitute a taboo for discussion, young people are hindered from actively seeking counseling regarding sexual health. Social ostracism and disease-associated stigma have created an attitude of negativity and shame in the minds of young people especially. This results in lack of knowledge about self-protection measures, leading to a silent spread of the disease.[9]

Adolescents are the age group at greatest risk for acquiring sexually transmitted diseases. Sexually transmitted disease intervention programs, based on behavioral change theories that emphasize on self-efficacy and motivational enhancement, may provide adolescents with the skills needed to change risk behavior patterns. School-based sexually transmitted disease programs can reach a majority of the ‘at-risk’ adolescent population. Community-based programs attempt to change community norms for a targeted high-risk population and are particularly helpful in reaching adolescents who are not in school. Finally, clinic-based interventions serve adolescents seeking healthcare, not only encouraging abstinence and safer sex practices for prevention of sexually transmitted disease, but also providing opportunities for early detection and treatment. All three have their advantages, but each may neglect a significant portion of the population at risk. Development of structured sexually transmitted disease intervention programs, utilizing school, community, and clinic settings merits further study.[10]

To assess the knowledge and beliefs of 428, 15–19-year-old girls and boys, they were interviewed regarding sexually transmitted infections (STIs) and HIV / AIDS. A community-based cross-sectional survey was conducted in 2002, in a rural district of Sindh province, Pakistan. The study concluded that rural adolescents need more knowledge regarding STIs including HIV / AIDS. There is a need to formulate strategies to raise the levels of awareness and knowledge among them regarding these conditions. These findings indirectly support the use of mass media and peer education strategies, to provide factual information to adolescents.[11]

Information about behavior, attitudes, and knowledge through regular surveys is essential to better understand the dynamics of the STI epidemic. This information is also important in assessing changes over time as a result of prevention efforts. In a cross-sectional survey conducted on adolescent girls in two urban higher secondary schools in South Delhi, India, to evaluate the adolescent school girls’ knowledge, perceptions, and attitude toward STIs / HIV and safer sex practice and sex education, and to explore their current sexual behavior, showed that 22% of young girls did not know that condom use could protect people from STI. It is important to note that among the school course elements that have generated the most controversy and debate in India, are discussions about homosexuality and information on the options of safer sex, including condom use and masturbation.[12]

Bridging the gap between knowledge and practice (particularly with respect to the use of condoms) has emerged as a major behavior change communication challenge to reducing adolescents’ vulnerability to STIs and unwanted pregnancies. The result of the study in Delhi, India, indicated that for adolescent students the Internet, media, friends, books, and magazines were the main sources of information regarding safe sex and HIV / STIs. Often students were confused or misinformed due to erroneous information received from these sources. Therefore evidence-based sex education must be a major strategy in school-based programs, with user friendly resources ready available to students. Unfortunately, many school students are exposed to sex and pornography through various television channels, cell phones, and Internet cafes. Educating adults and children is essential under these non-regulated conditions. According to AIDS activists, sex education helps to make students cautious against the dangers of experimenting with sex at a young age, sensitizing them and also warning them about the potential exposure to deadly diseases. Many parents are hesitant to talk about sex with their teenage children at home; even mothers hesitate to talk to daughters about something as simple as menstruation. Moreover, widespread illiteracy underlines the importance of being able to talk about sexuality comfortably in a gathering or congregation. Policymakers believe that many people will be critical of moves to implement sex education to young people, and therefore, may seek political advantage by promoting traditional values.[12]

The strength of the study is that the findings revealed that adolescent high school girls in this part of India are in special need of psychosomatic health intervention on correct knowledge and positive attitude.

The limitation of the study is that it needed to be repeated at regular intervals to monitor the problem by integrated health service research.

During interactive sessions, the study participants suggested that the school should provide information about sexuality from the lower grades of secondary schools.

## CONCLUSION

Our study revealed some unknown part of adolescent psychology in this part of India. At the crossroad of childhood and mature adulthood many diversions come in the way of rational thinking in adolescents. They go for experimentation of different types. Many a times the caregivers are unable to explore their needs, demands, and attitude regarding knowledge on the ongoing and inevitable physical changes and psychological developments. Moreover, addiction, and common ailments need to be addressed in the light of Life skill Education programs for Adolescents recently launched. This in-depth study was intended to verify the desired modification level as well as with a view to explode any myth regarding adolescent sex education. Although some sort of sex education is part of many schools’ th curriculum, it remains a th controversial topic in several countries, as to how much and at what age schoolchildren should be taught about contraception or th safer sex, th masturbation, and whether th moral education should be included or excluded In the th United States in particular, the topic is the subject of much contentious debate. Chief among the controversial points is whether sexual freedom for minors is valuable or detrimental, and also whether instruction about th condoms and th birth control pills will reduce or increase out-of-wedlock or th teenage pregnancies and STDs. The existence of th AIDS has given a new sense of urgency to the topic of sex education. In many th African nations, where AIDS is at epidemic levels, sex education is seen by most scientists as a vital strategy for preserving the health of citizens.
